# Diffusion tensor imaging and disability progression in multiple sclerosis: A 4‐year follow‐up study

**DOI:** 10.1002/brb3.1194

**Published:** 2018-12-26

**Authors:** Marcin Kolasa, Ullamari Hakulinen, Antti Brander, Sanna Hagman, Prasun Dastidar, Irina Elovaara, Marja‐Liisa Sumelahti

**Affiliations:** ^1^ Faculty of Medicine and Life Sciences Tampere University Tampere Finland; ^2^ Department of Radiology, Medical Imaging Center of Pirkanmaa Hospital District Tampere University Hospital Tampere Finland; ^3^ Faculty of Biomedical Sciences and Engineering Tampere University of Technology Tampere Finland; ^4^ Department of Medical Physics, Medical Imaging Center Tampere University Hospital Tampere Finland

**Keywords:** diffusion tensor imaging, longitudinal study, multiple sclerosis

## Abstract

**Objectives:**

Diffusion tensor imaging (DTI) is sensitive technique to detect widespread changes in water diffusivity in the normal‐appearing white matter (NAWM) that appears unaffected in conventional magnetic resonance imaging. We aimed to investigate the prognostic value and stability of DTI indices in the NAWM of the brain in an assessment of disability progression in patients with a relapsing‐onset multiple sclerosis (MS).

**Methods:**

Forty‐six MS patients were studied for DTI indices (fractional anisotropy (FA), mean diffusivity (MD), radial (RD), and axial (AD) diffusivity) in the NAWM of the corpus callosum (CC) and the internal capsule at baseline and at 1 year after. DTI analysis for 10 healthy controls was also performed at baseline. Simultaneously, focal brain lesion volume and atrophy measurements were done at baseline for MS patients. Associations between DTI indices, volumetric measurements, and disability progression over 4 years were studied by multivariate logistic regression analysis.

**Results:**

At baseline, most DTI metrics differed significantly between MS patients and healthy controls. There was tendency for associations between baseline DTI indices in the CC and disability progression (*p* < 0.05). Changes in DTI indices over 1 year were observed only in the CC (*p* < 0.008), and those changes were not found to predict clinical worsening over 4 years. Clear‐cut association with disability progression was not detected for baseline volumetric measurements.

**Conclusion:**

Aberrant diffusivity measures in the NAWM of the CC may provide additional information for individual disability progression over 4 years in MS with the relapsing‐onset disease. CC may be a good target for DTI measurements in monitoring disease activity in MS, and more studies are needed to assess the related prognostic potential.

## 1 INTRODUCTION

In multiple sclerosis (MS), demyelination and axonal injury in the central nervous system are responsible for neurological disability. Conventional magnetic resonance imaging (MRI) detecting T1 and T2 focal brain lesions is not specific to the underlying pathology, and it lacks sensitivity to the microstructural diffuse damage in the normal‐appearing white matter (NAWM) (Filippi, Absinta, & Rocca, 2013). Conventional MRI markers correlate only moderately with clinical disability (Tintore et al., 2015), and their prognostic value in the assessment of disability progression in definite MS is limited (Filippi et al., 2013). Consequently, brain atrophy that has been related to long‐term disability in MS (De Stefano et al., 2016) expresses the underlying pathological processes only nonspecifically. Confounding factors, such as disease‐modifying therapies and causes unrelated to MS, complicate interpretation of MRI markers and atrophy in clinical practice (Kaunzner & Gauthier, 2017; Wattjes et al., 2015).

Diffusion tensor imaging (DTI) quantifies the magnitude and direction of water diffusion, and it is sensitive to diffuse microstructural abnormalities in the brain that appears unaffected on conventional MRIs (Rovaris et al., 2005). DTI‐derived metrics, including fractional anisotropy (FA), mean diffusivity (MD), radial (RD), and axial (AD) diffusivities, seem to provide a better specificity to demyelination and axonal injury than conventional MRIs (Sun et al., 2006). Increased MD and decreased FA in the NAWM of different brain regions, including the corpus callosum (CC), have been typically detected in MS (Banaszek, Bladowska, Pokryszko‐Dragan, Podemski, & Sasiadek, 2015; Preziosa et al., 2011; Sigal, Shmuel, Mark, Gil, & Anat, 2012). However, inconsistent results regarding the correlation between disability and DTI indices in the CC and the pyramidal tract have been reported in cross‐sectional studies using different methods of DTI analysis and clinical scales of disability (Lin, Yu, Jiang, Li, & Chan, 2007; Llufriu et al., 2012; Pokryszko‐Dragan et al., 2018; Roosendaal et al., 2009; Tortorella et al., 2014). The correlation between RD and secondary progression in MS has been observed in a 50‐year clinical follow‐up study indicating the potential role of DTI in the prediction of outcomes in MS (Andersen et al., 2018).

Previously, decreased FA and increased RD mostly in the CC of MS were observed in a 2‐year longitudinal study (Harrison et al., 2011). In contrast, no changes in diffusivity were observed in the NAWM of MS over 2–4 years (Ontaneda et al., 2017; Rashid et al., 2008). Moreover, few studies with a short (1–2 years) follow‐up have applied a regional and whole‐brain DTI analysis to longitudinal measurements of diffusivity aiming to evaluate the prognostic value of DTI in the assessment of disability progression in MS (Rashid et al., 2008; Samann et al., 2012; Schmierer et al., 2004). In one of these studies, the increase of MD in the white matter of frontal lobe over 1 year was associated with clinical impairment in primary‐progressive MS (Schmierer et al., 2004), while in another study, in early relapsing‐remitting MS, no diffusivity changes were detected over 2 years (Rashid et al., 2008).

The investigation of the prognostic value of DTI in this cross‐sectional and 4‐year longitudinal study aims to assess white matter diffusion change and its stability in the relapsing‐onset MS cohort considering the variable rate of disease progression.

## 2 MATERIALS AND METHODS

The study was approved by the local ethics committee in the Hospital District of Pirkanmaa (R05157). All subjects provided informed written consent.

### 2.1 Subjects

In total, 56 individuals, 46 patients with relapsing‐onset MS, and 10 healthy subjects were enrolled in this 4‐year follow‐up study (between 2006 and 2012) at the Tampere University Hospital, Finland. The MS diagnosis was based on the revised McDonald criteria from 2005 (Polman et al., 2005) and the disease course classification on Lublin and Reingold criteria (Lublin et al., 2014). The inclusion criteria were a diagnosis of relapsing‐remitting MS (RRMS) or secondary‐progressive MS (SPMS), no steroid treatment at least 8 weeks before clinical and radiological assessments, and an Expanded Disability Status Scale (EDSS) score both at the study entry and after 4 years. Healthy subjects consisted of five females and five males, and the mean age of the subjects was 39.7 years (range 26–61). Healthy subjects were recruited from the hospital staff or their relatives with no history of neurological or psychiatric illness.

During the follow‐up, MS patients underwent a clinical examination by the same neurologist at baseline and annually for 4 years (in total five examinations). Clinical progression was determined as the difference between the baseline EDSS and EDSS 4 years after the baseline. Progression of disability during the follow‐up was defined as an EDSS score increase ≥1.0 when the baseline EDSS was <6.0 or an increase of EDSS ≥ 0.5 when the baseline EDSS ≥ 6.0, and these subjects were assigned to a progression group (Rovaris et al., 2003). All the other patients were included in the stable group.

### 2.2 MR imaging acquisition

MRI volumetry included T1 and FLAIR brain lesion volume and brain atrophy measurements, and it was carried out at baseline for 42 MS patients. DTI in 46 cases was performed at baseline and 1 year after the baseline visit. Healthy controls were assessed with DTI at baseline.

The patients underwent MRI on the same day as a clinical examination. The patients and controls underwent a whole‐brain imaging by using a 1.5‐Tesla MR scanner (Magnetom Avanto SQ, Siemens Medical Solutions, Erlangen, Germany), and the MRI acquisition and protocol were as follows: T1‐weighted header followed by an axial three‐dimensional (3D) T1‐weighted magnetization prepared rapid gradient echo (MPRAGE), 3D T2‐weighted turbo spin echo, fluid‐attenuated inversion recovery (FLAIR), T1‐weighted spin echo with magnetization transfer contrasts, multidirectional diffusion‐weighted echo‐planar imaging, and gadolinium‐enhanced T1‐weighted MPRAGE when needed. The DTI protocol consisted of a single‐shot spin‐echo‐based echo‐planar diffusion‐weighted imaging with three averages and 12 gradient encoding directions, with b values of 0 and 1,000 s/mm^2^. The imaging parameters are presented in Table 1.

**Table 1:** Imaging parameters

	Axial T1WI	Axial FLAIR	Axial DTI
Slice thickness (mm)	0.9	5	5
Interslice gap (mm)	0	0	1.5
Field of view (mm)	230 × 230	230 × 230	230 × 230
Matrix	256 × 256	256 × 256	128 × 128
Echo time (ms)	4.2	100	96
Repetition time (ms)	1,160	8,500	3,500
Inversion time (ms)	600	2,500	

Note

DTI: diffusion tensor imaging; FLAIR: fluid‐attenuated inversion recovery; T1WI: T1‐weighted imaging.

### 2.3 MR imaging postprocessing

**Figure 1:**
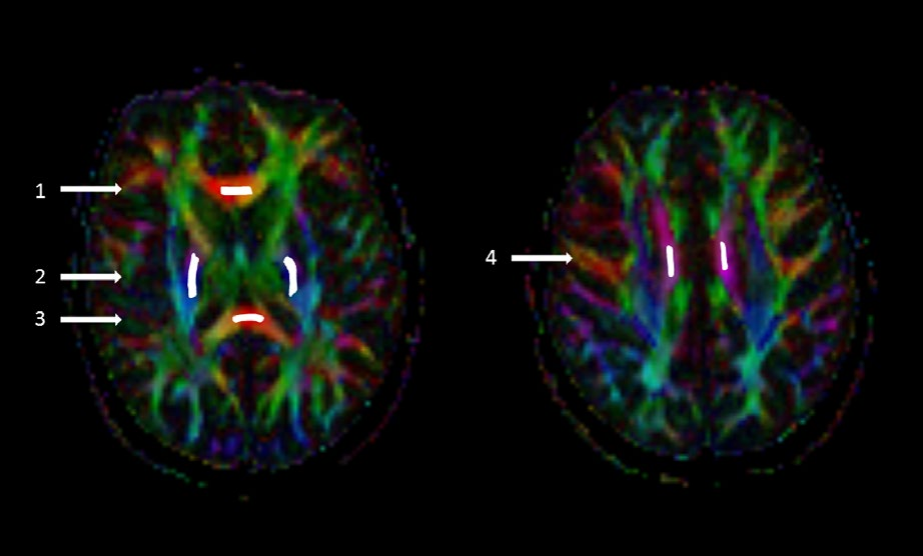
Freehand ROI placement on the color‐coded fractional anisotropy axial maps. (1) Genu of the corpus callosum (size of ROI means 26 mm^2^, range 13–71), (2) posterior limb of the internal capsule (48 mm^2^, 13–81), (3) splenium of the corpus callosum (32 mm^2^, 13–81), (4) body of the corpus callosum (26 mm^2^, 19–84). Pixel size 1.8 × 1.8 mm

The DTI data were analyzed with commercial Neuro 3D software (Siemens Healthcare, Malvern, USA) at an offline workstation. Multidirectional diffusion data were assessed visually for the presence of distortions and artifacts. There were no significant eddy current distortions due to the diffusion gradients. Six freehand regions of interest (ROI) of approximately 26–48 mm^2^ (depending on the anatomical region) were positioned on the left and right posterior limbs of the internal capsule (IC), CC genu, left and right CC body, and CC splenium (Figure 1). The ROIs were manually placed exactly the same way at both time points on axial images of the color‐coded FA maps and were automatically transferred on the MD, eigenvalues, and non‐diffusion‐weighted b_0_ maps. The ROIs were centered on the anatomical structure in the most homogeneous area, with guidance from conventional T2 images to exclude focal lesions from the ROI and partial volume effect from border areas. The size of ROI was reduced if a focal lesion was identified in the ROI. The difference in ROI size between the baseline and 1‐year follow‐up was very small, <9% (range 1.8%–8.8%) in all ROIs. The values of the following DTI parameters were obtained: FA, MD, AD, and RD.

The whole brain volume of the T1 hypointense, FLAIR hyperintense lesions, and brain parenchymal fraction (BPF) were assessed blindly using the semi‐automatic segmentation software Anatomatic™ 2.23 (Heinonen et al., 1998) by the same reader. BPF was defined as a ratio of brain parenchymal volume to the total volume within the brain surface contour (Rudick, Fisher, Lee, Simon, & Jacobs, 1999).

### 2.4 Statistical analysis

Means and standard deviations were given for normally distributed variables and medians and ranges for skewed distributed data. For the demographic and volumetric data, groups were compared using independent sample *t* tests for normally distributed continuous variables and Mann–Whitney *U* tests for skewed distributed continuous variables. Spearman's rank correlations were determined for correlations between clinical and MRI parameters. The Wilcoxon test was used to perform comparisons between DTI values at baseline and 1 year. To investigate association between DTI metrics, volumetric measurements, and disability progression over 4 years, a series of logistic regression models were created. The presence or absence of disability progression was used as a dependent variable in all models. In logistic regression Model 1, the age and time from the onset (first symptoms) to baseline were set as covariates. In Model 2, the covariates were as follows: sex, disease duration (time from MS diagnosis to baseline), baseline EDSS, number of relapses up to 3 years preceding the baseline, immunomodulatory medication status, and volumetric measurements (T1/FLAIR lesion volume, BPF). A resulting odds ratio (OR) is given with 95% confidence interval (CI), and the *p*‐value <0.05 was considered statistically significant. The Bonferroni‐corrected *p*‐values for six comparisons (*p* < 0.008) were also investigated in the analyses concerning DTI. A statistical analysis was performed using SPSS Statistics for Windows version 22 (IBM Corp., Armonk, NY, USA).

## 3 RESULTS

### 3.1 Clinical and radiological assessment at baseline and over the follow‐up

In total, 22 of 46 (48%) patients showed disability progression over 4 years. The mean age of patients at baseline was 39.6 years (range 18–61). The demographic and clinical characteristics are summarized in Table 2. Seven patients had one demyelinating plaque in the IC, four patients had one demyelinating plaque in the CC, and one patient had several plaques in the CC.

**Table 2:** Demographic, clinical and radiological data for MS patients

	Whole group	Stable group	Progression group	*p*‐Value^a^
No. of patients	46	24	22	
Female:male	31:15	17:7	14:8	0.6
Mean age at baseline, years, mean (range)	39.6 (18–61)	39.1 (20–61)	40.2 (18–58)	0.3
Median time from onset symptom to baseline, years (range)	9 (0.7–32.2)	7.6 (1.4–32.2)	12.3 (0.7–31.2)	0.6
Median disease duration, years (range)	4.2 (0–31.2)	2.3 (0–27.2)	5.9 (0–31.2)	0.1
EDSS, median (range)				
Baseline	2 (0–7)	1.5 (0–6)	3.0 (0–7)	0.2
Year 1	2 (0–7.5)	1.5 (0–6)	3.5 (0–7.5)	
Year 2	2.5 (0–8)	1.5 (0–6)	5.5 (0–8)	
Year 3	2 (0–8)	1.5 (0–6)	5.5 (0–8)	
Year 4	2 (0–8)	1.5 (0–6)	6.0 (1–8)	**<0.001**
Difference between EDSS over 4 years, median (range)	0.5 (–1.5 to 4)	0 (0.5 to −1.5)	1.5 (0.5–4)	
No. of relapses up to three years before baseline, no. of patients (%)				
0	15 (33)	5 (21)	10 (45)	0.07
1–2	24 (52)	14 (58)	10 (45)	
3–5	7 (15)	5 (21)	2 (10)	
No. of relapses during the follow‐up, no. of patients (%)				
0	24 (52.2)	12 (50)	12 (54.5)	1.00
1–2	12 (26.1)	7 (29.2)	5 (22.7)	
3–6	10 (21.7)	5 (20.8)	5 (22.7)	
Duration of treatment at baseline, months, median (range)	18.5 (1–122)	18.5 (1–70)	15.5 (1–122)	0.9
Treatment at baseline, no. of patients (%)^b^	18 (39)	12 (50)	6 (27)	0.2
Treatment at the end of the follow‐up, no. of patients (%)^b^	20 (43.4)	12 (50)	8 (36)	0.3
T1 brain lesion load at baseline cm^3^, median (range)^c^	1.7 (0.1–28.5)	1 (0.1–28.5)	2.2 (0.1–14.7)	0.1
FLAIR brain lesion load at baseline cm^3^, median (range)^c^	5.8 (1–39)	2.8 (1–39)	8.2 (1–33)	**0.03**
Brain parenchymal fraction at baseline, median (range)^c^	0.72 (0.6–0.81)	0.73 (0.64–0.8)	0.68 (0.6–0.81)	0.2

Note

EDSS, Expanded Disability Status Scale; Range was defined as minimum and maximum values.

a Comparison between stable versus progression groups, Mann‐Whitney *U* test for median values, *t* test for mean values, and chi‐square test for descriptive data; in bold, *p* < 0.05.

b First‐line treatment (beta‐interferon, glatiramer acetate).

c Values calculated for 42 MS patients (23 patients in stable group, 19 patients in progression group); there were no significant differences regarding clinical and demographic data between group of patients with DTI (*n* = 46) and the volumetric analysis.

Incidental findings in the brain white matter were found in four healthy subjects from control group; three subjects had one to two punctate white matter hyperintensities, one subject had several punctate white matter hyperintensities, and none of healthy subjects presented clinical signs of demyelinating disease.

In MS, compared to healthy subjects, the strongest differences (*p* < 0.001) were found in the CC for FA, MD, and RD (Supporting Information Figure S1).

The FLAIR lesion volumes were significantly higher (*p* < 0.05) in the disability progression group compared to the stable disability group (Table 2). No significant correlations were found between baseline DTI and age, disease duration, baseline EDSS, and number of relapses before baseline (data not shown).

At baseline, significant correlations (*p* < 0.008, *r* > 0.4) were found between MRI volumetric measurements and DTI indices. The strongest correlations were found in the CC genu between the T1 brain lesion volume and FA (*p* = 0.001, *r* = −0.48), MD (*p* < 0.001, *r* = 0.52), RD (*p* < 0.001, *r* = 0.52) and between FLAIR lesion volume and FA (*p* < 0.001, *r* = −0.6), MD (*p* < 0.001, *r* = 0.54), and RD (*p* < 0.001, *r* = 0.6). Regarding brain atrophy, the strongest correlations were found between BPF and RD (*p* = 0.002, *r* = −0.46) in the right CC body, RD (*p* = 0.007, *r* = −0.41) in the left CC body, MD (*p* = 0.004, *r* = −0.44) and AD (*p* = 0.001, *r* = −0.49) in the right IC, and MD (*p* < 0.001, *r* = −0.53) and AD (*p* = 0.002, *r* = −0.47) in the left IC (Supporting Information Table S1).

During the 1‐year follow‐up, FA significantly (*p* < 0.05) increased in 4/6 ROIs, and RD decreased in 4/6 ROIs (CC genu, body, and the CC splenium). AD showed a significant increase in 3/6 ROIs (the CC genu, CC body). The results remained significant except for RD in the CC genu and AD in left CC body after the Bonferroni corrections (*p* < 0.008). In the IC, the changes were nonsignificant (Table 3).

**Table 3:** DTI indices at baseline and after 1 year of the follow‐up in MS patients

Relapsing‐onset MS patients, *n* = 46										
DTI metrics	Baseline			Year 1			Annual change			*p*‐Value^a^
	Median	Min	Max	Median	Min	Max	Median	Min	Max	
Corpus callosum genu										
FA	0.78	0.48	0.88	0.81	0.48	0.93	0.03	−0.08	0.14	**<0.001**
MD	0.80	0.62	1.31	0.82	0.67	1.07	−0.01	−0.34	0.20	0.891
AD	1.73	1.47	2.25	1.84	1.40	2.23	0.10	−0.62	0.43	**0.006**
RD	0.34	0.17	0.84	0.32	0.12	0.66	−0.04	−0.25	0.14	**0.009**
Corpus callosum body right										
FA	0.56	0.30	0.87	0.68	0.32	0.89	0.06	−0.17	0.30	**<0.001**
MD	0.83	0.58	1.08	0.81	0.70	1.11	0.01	−0.17	0.24	0.797
AD	1.47	1.07	1.84	1.63	1.13	1.95	0.12	−0.37	0.65	**0.003**
RD	0.53	0.20	0.92	0.44	0.20	0.75	−0.08	−0.33	0.11	**<0.001**
Corpus callosum body left										
FA	0.58	0.29	0.85	0.68	0.37	0.88	0.10	−0.18	0.31	**0.001**
MD	0.82	0.67	1.39	0.83	0.68	1.11	0.01	−0.42	0.24	0.589
AD	1.46	1.06	2.08	1.64	1.08	2.00	0.12	−0.37	0.65	**0.027**
RD	0.52	0.23	1.14	0.45	0.20	0.74	−0.09	−0.40	0.15	**<0.001**
Corpus callosum splenium										
FA	0.79	0.52	0.94	0.82	0.58	0.94	0.02	−0.07	0.20	**0.004**
MD	0.75	0.56	1.28	0.75	0.58	1.11	−0.02	−0.20	0.21	0.215
AD	1.67	1.23	2.13	1.69	1.42	2.07	0.04	−0.27	0.30	0.157
RD	0.28	0.11	0.86	0.25	0.09	0.69	−0.04	−0.31	0.14	**0.003**
Internal capsule right										
FA	0.72	0.62	0.83	0.71	0.55	0.85	0.00	−0.16	0.07	0.304
MD	0.74	0.66	0.79	0.74	0.67	0.83	0.01	−0.05	0.10	0.245
AD	1.46	1.33	1.73	1.45	1.27	1.80	0.00	−0.13	0.14	0.743
RD	0.36	0.24	0.47	0.37	0.23	0.52	0.00	−0.08	0.18	0.345
Internal capsule left										
FA	0.71	0.47	0.80	0.71	0.49	0.83	0.01	−0.18	0.32	0.814
MD	0.73	0.66	0.87	0.73	0.66	0.84	0.01	−0.06	0.07	0.092
AD	1.46	1.25	1.69	1.48	1.25	1.83	0.03	−0.20	0.45	0.068
RD	0.35	0.26	0.61	0.35	0.25	0.58	0.01	−0.27	0.15	0.566

Note

Annual change is defined as difference between median DTI value at 1 year and median DTI value at baseline.

DTI: diffusion tensor imaging; FA: fractional anisotropy; MD: mean diffusivity (×10^−3^ mm^2^/s); axial diffusivity (×10^−3^ mm^2^/s); radial diffusivity (×10^−3^ mm^2^/s).

a *p*‐Value for Wilcoxon test; in bold, *p* < 0.05.

No group differences existed regarding DTI change over 1 year in any ROIs between disability progression and stable groups (data not shown).

To assess the intra‐observer repeatability of DTI measurements, the intraclass correlations (ICCs) were calculated for 20 patients. The same observer (U.H.) repeated the measurements for the same scans with a time interval of approximately 3 months. In all ROIs, the ICCs were good and excellent and were 0.77–0.98 (mean 0.91) for FA, 0.75–0.96 (mean 0.84) for MD, 0.64–0.93 (mean 0.85) for AD, and 0.85–0.95 (mean 0.91) for RD.

### 3.2 Association between MRI markers and disability progression

In logistic regression Model 1 with covariates of age and time from the onset to baseline (Table 4), a lower baseline FA and higher RD in the CC genu, right CC body, and the CC splenium were associated with disability progression (*p* ˂ 0.05). Moreover, a higher baseline MD in the right CC body and higher MD and AD in the CC splenium were associated with disability progression. The results did not remain significant after the Bonferroni corrections. There were no significant associations between baseline DTI indices in the IC and disability progression over the follow‐up. The age and symptom time had no effect in any of the analyzed ROIs.

**Table 4:** Relationship of baseline DTI metrics with disability progression measured by EDSS increase over the 4‐year follow‐up

DTI metrics	Stable group *n* = 24			Progression group *n* = 22			*p*‐Value^a^	Odds ratio	95% CI	
	Median	Min	Max	Median	Min	Max				
Corpus callosum genu										
FA	0.81	0.52	0.88	0.74	0.48	0.88	**0.04**	0.00	0.00	0.61
MD	0.80	0.62	1.21	0.83	0.67	1.31	0.06	1.05	1.00	1.10
AD	1.70	1.47	2.07	1.76	1.55	2.25	0.36	1.02	0.98	1.06
RD	0.28	0.17	0.80	0.37	0.17	0.84	**0.04**	1.05	1.00	1.09
Corpus callosum body right										
FA	0.67	0.40	0.87	0.52	0.30	0.77	**0.01**	0.00	0.00	0.24
MD	0.80	0.58	1.07	0.87	0.69	1.08	**0.04**	1.08	1.00	1.15
AD	1.51	1.07	1.79	1.39	1.18	1.84	0.25	0.98	0.95	1.01
RD	0.46	0.20	0.73	0.62	0.30	0.92	**0.01**	1.07	1.02	1.12
Corpus callosum body left										
FA	0.66	0.38	0.85	0.53	0.29	0.82	0.07	0.02	0.00	1.37
MD	0.81	0.67	1.32	0.84	0.70	1.39	0.12	1.03	0.99	1.08
AD	1.50	1.19	2.08	1.42	1.06	2.04	0.53	0.99	0.97	1.02
RD	0.46	0.23	0.93	0.58	0.30	1.14	**0.04**	1.04	1.00	1.08
Corpus callosum splenium										
FA	0.82	0.63	0.89	0.76	0.52	0.94	**0.04**	0.00	0.00	0.76
MD	0.71	0.56	0.95	0.79	0.68	1.28	**0.01**	1.13	1.03	1.23
AD	1.62	1.23	1.87	1.80	1.50	2.13	**0.01**	1.08	1.02	1.14
RD	0.25	0.16	0.50	0.35	0.11	0.86	**0.02**	1.07	1.01	1.14
Internal capsule right										
FA	0.72	0.62	0.78	0.72	0.62	0.83	0.89	1.01	0.89	1.14
MD	0.72	0.66	0.79	0.75	0.66	0.78	0.14	1.13	0.96	1.33
AD	1.45	1.33	1.61	1.49	1.34	1.73	0.16	1.05	0.98	1.12
RD	0.35	0.29	0.47	0.36	0.24	0.46	0.78	1.02	0.90	1.15
Internal capsule left										
FA	0.71	0.47	0.80	0.71	0.58	0.80	0.49	1.03	0.94	1.13
MD	0.71	0.66	0.87	0.74	0.66	0.80	0.15	1.12	0.96	1.32
AD	1.42	1.25	1.63	1.48	1.30	1.69	0.05	1.07	1.00	1.15
RD	0.35	0.26	0.61	0.36	0.31	0.48	0.86	0.99	0.90	1.09

Note

DTI: diffusion tensor imaging; FA: fractional anisotropy; MD: mean diffusivity (×10^−3^ mm^2^/s); axial diffusivity (×10^−3^ mm^2^/s); radial diffusivity (×10^−3^ mm^2^/s); EDSS: Expanded Disability Status Scale.

a *p*‐Value for logistic regression adjusted for age and duration of symptoms for prediction of EDSS progression over the 4‐year follow‐up; in bold, *p* < 0.05.

In Model 2, which contained baseline EDSS and relapse number before baseline, an association between DTI and disability progression disappeared in the CC genu, body, and the splenium regrading several diffusivity parameters; however, none of these explanatory variables reached statistical significance (Supporting Information Tables S2 and S3). Medication, disease duration, and sex had no effect on disability progression (data not shown). T1, FLAIR, and BPF were not explanatory for disability progression (Supporting Information Table S4). However, the association between disability progression and DTI disappeared in the CC genu, and statistical power slightly decreased in the other CC areas in the models, including FLAIR lesion volume and BPF. The T1 lesion volume had no effect in any regression model (data not shown).

DTI change over 1 year did not relate to disability progression over 4 years in any ROIs (data not shown).

## 4 DISCUSSION

The prognostic assessment of clinical disability accumulation by using conventional MRI is still suboptimal (Filippi et al., 2013), where additional challenges concern the individual and heterogenous disability progression. In the present study, the relapsing‐onset MS patient cohort showed altered DTI indices at baseline compared to healthy controls, especially in the CC and to a lesser degree in the IC. The anatomical location of the observed differences may indicate that DTI is sensitive to microstructural abnormalities occurring in the NAWM tracts responsible for cognitive and locomotor functions. Our finding corroborates other reports showing that AD is less affected when compared to RD in the CC and the pyramidal tract, including IC (Henry, Oh, Nelson, & Pelletier, 2003; Lin et al., 2007; Roosendaal et al., 2009).

In the NAWM of MS, FA is typically decreased, whereas MD is increased, expressing the loss of white matter tracts directionality and the increase in overall water diffusivity, respectively (Alexander, Lee, Lazar, & Field, 2007). Increased RD, a measure of perpendicular diffusivity to the fibers, is usually linked to demyelination (Fink et al., 2010). Diffusion parallel to the fibers, that is, AD, a marker of axonal integrity, is typically decreased and correlates clearly with axonal damage at the early stages of MS. At the chronic stage of MS, AD may conversely increase, representing the confounding effect of reparative processes, such as gliosis and cellular infiltration (Aung, Mar, & Benzinger, 2013). In this context, the nonsignificant difference between healthy controls and MS patients in our study may result from different directions of change in AD representing competing pathological processes at different progression stages in MS. The correlation between baseline brain lesion volume, brain atrophy, and DTI measurements in our MS group suggests that diffusivity abnormalities may be secondary to progression, both Wallerian degeneration of the axons passing through remote macroscopic brain lesions (Ge et al., 2004; Lin et al., 2007) and brain atrophy due to the partial volume effect within voxels (Roosendaal et al., 2009).

The main observation in our study is the tendency for baseline DTI metrics’ association in the CC with disability progression over 4 years with the most consistent and stable correlation observed in the CC splenium. We observed an increased baseline AD and RD, indirectly representing axonal integrity and demyelination, which is associated with disability progression even after correcting for focal lesion volume. This result corroborates observations in a previous study analyzing only FA maps where decreased FA in the CC splenium in primary‐progressive MS (Bodini et al., 2013) was associated with EDSS progression over 5 years; however, longitudinal stability of DTI indices has not been analyzed in this study. As we investigated longitudinal changes in both AD and RD indices, which are more specifically related to MS pathology, we can speculate that inflammatory activity and axonal degeneration are responsible for clinical worsening in our MS cohort. Similar to our results, increased RD in the CC body has been associated with motor impairment expressed by the 9‐hole peg test (NHPT) in a 1‐year follow‐up study with a small number (*n* = 22) of patients with RRMS (Kern, Sarcona, Montag, Giesser, & Sicotte, 2011). Moreover, a histogram‐based analysis revealed a correlation between whole‐brain diffusivity alterations and disability progression expressed by the MS Functional Composite Scale over 1 year (Samann et al., 2012). The significance of our observation is strengthened by the fact that axonal degeneration, represented here by increased AD, is mainly responsible for sustained disability in MS (Tallantyre et al., 2010). The reason why the most stable correlation between DTI and disability progression was observed in the CC splenium of our study cohort might be related to thin axons that are densest in the splenium and their preferential susceptibility to injury in MS, as also suggested by others (Ciccarelli et al., 2003). As the CC body is a thin anatomical structure, the partial volume effect from cerebrospinal fluid may influence the results of DTI measurements in the region we observed. ROI‐based methodology is sensitive to the change in DTI parameters, avoids postprocessing calculation errors, and is suitable for investigating well‐defined brain structures such as CC and IC (Snook, Plewes, & Beaulieu, 2007). Good reproducibility of DTI measurements in our present and previous studies (Brander et al., 2010; Hakulinen et al., 2012; Kolasa et al., 2015), along with coherent fibers in the white matter tracts of the IC and the CC, suggests that diffusivity abnormalities, as observed here, may be related to white matter pathology rather than method‐based variability or crossing fibers within a voxel (Wheeler‐Kingshott & Cercignani, 2009).

The 1‐year longitudinal DTI analysis revealed a significant change of DTI metrics in the CC but not in the IC. In this cohort with active MS (Lublin et al., 2014), we observed an increase instead of the expected decrease of FA in the CC. This increase was driven by increased AD and decreased RD in the CC genu and the body and decreased RD in the CC splenium. Due to a short radiological follow‐up with only two MRI examinations, we cannot fully determine the sustained changes in DTI parameters. A longitudinal DTI study with shorter interval MRI examinations would be more appropriate to evaluate the temporal changes in diffusivity (Tian et al., 2012). Moreover, without healthy controls in the longitudinal analysis, we cannot clearly assess pathophysiological processes involved in temporal DTI changes observed here in the CC. Similar to our observation, serial DTI study using tractography showed significant longitudinal change in DTI metrics in the supratentorial brain and the CC of the MS cohort with different disease phenotypes (Harrison et al., 2011). However, such temporal DTI evolution was not observed in a recent ROI‐based MS study including natalizumab‐treated patients (Ontaneda et al., 2017). In another study in early RRMS with a 2‐year follow‐up, the rate of change in diffusivity characteristics assessed by a histogram‐based whole‐brain analysis did not correlate with disability progression expressed by an EDSS increase, which confirms our results (Rashid et al., 2008). Conversely, the association between diffusivity in the frontal NAWM and disability as measured by the MS Functional Composite Scale has been found in primary‐progressive MS (Schmierer et al., 2004). Thus, inconsistent results observed in previous studies may relate to technical differences, intrinsic heterogeneity of MS (Barone et al., 2018) and MS cohorts, and different clinical scales used in disability evaluation in MS.

Altogether, the results of our longitudinal study suggest that DTI is a sensitive tool in monitoring diffuse abnormalities responsible for disability accumulation, and CC may be a good target for DTI analysis. We believe that an assessment of the prognostic value of DTI in an MS cohort with variable clinical characteristics such as ours which is typically encountered in everyday practice has practical value, as suggested by others (Harrison et al., 2011). Moreover, changes in RD observed here may play an important role in monitoring immunomodulatory treatment effects because the attenuation of inflammatory demyelination is the main target of current MS therapies. This statement is supported by the results of the study by Fox et al., where DTI abnormalities indicating remyelination have been observed after starting natalizumab treatment (Fox et al., 2011).

We did not observe any associations in the IC between baseline DTI and disability progression. Moreover, no longitudinal changes in DTI metrics were observed in the IC, although significant differences related to DTI between healthy controls and the MS group were already observed. This result indicates that diffusivity abnormalities may already exist in the IC, but they progress at different rates, and image disability progression distinctly than in the CC (Ge et al., 2004). Our finding is supported by studies where no correlation between DTI indices in the corticospinal tract and disability progression expressed by an EDSS increase has been observed (Fritz, Keller, Calabresi, & Zackowski, 2017; Lin et al., 2007). Conversely, such correlation between DTI parameters and EDSS has been previously reported in cross‐sectional studies (Daams et al., 2015; Tovar‐Moll et al., 2015).

Our results corroborate the observed lack of clear‐cut association between the T1/T2 brain lesion load, brain atrophy, and disability progression expressed by EDSS change in other follow‐up studies for over 2 years in relapsing MS (Enzinger et al., 2011; Tiberio et al., 2005). Although volumetric measurements did not clearly correlate with disability progression in our study, the FLAIR lesion volume and BPF showed some effect and modified the correlation between DTI and disability progression. In contrast to our results, association between short‐term physical worsening, T2 brain lesion load (Gauthier et al., 2007; Moodie et al., 2012), and brain atrophy has been reported elsewhere (Minneboo et al., 2008; Samann et al., 2012). These discordant results suggest that the focal brain lesion load and brain atrophy may have additional impact on disability accumulation in relapsing‐onset MS. The lack of significant correlation here may be limited by a small number of cases in the study cohort, where disease activity and disability progression were variable. Other limitations in our inferences may result from the fairly gross nature of total EDSS in a situation where there is a need to evaluate subtle changes in motor functions during a short observation period.

In conclusion, our results among others suggest that diffusivity abnormalities exist in relapsing‐onset MS patients; however, their dynamic change over time is different with respect to anatomical location. Additionally, diffusivity metrics in the normal‐appearing CC may be associated with disability accumulation in relapsing‐onset MS and suggest the crucial role of the CC in monitoring disease progression. Given its high sensitivity in detecting diffuse brain abnormalities, DTI indices may serve as a potential biomarker of disease progression; however, method standardization is needed. Moreover, stability and sensitivity to underlying pathology of DTI metrics have to be confirmed in longitudinal studies (Wattjes et al., 2015). A combination of diffusion measures with other findings from conventional MRI may provide complementary information on different types of pathological damage in MS.

## CONFLICT OF INTEREST

We declare that we have no conflict of interest.
