# Supplementary figures and images for: Diffusion tensor imaging and disability progression in multiple sclerosis: A 4‐year follow‐up study
Source: Brain Behav. 2018 Dec 26;9(1):e01194. doi: 10.1002/brb3.1194 (PMC6346728; doi:10.1002/brb3.1194)

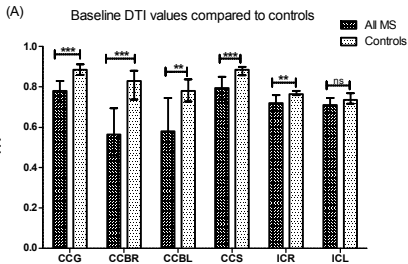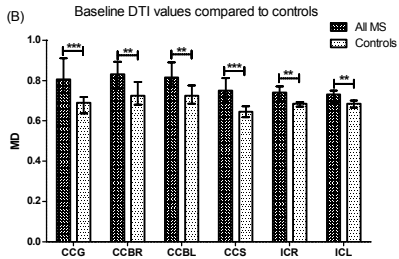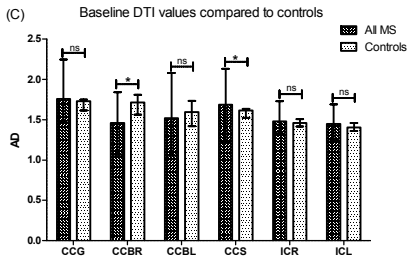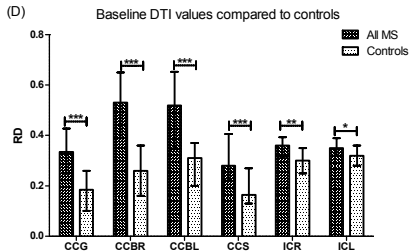

Supplement: Supplementary file 1 [file BRB3-9-e01194-s001.pdf]
